# Site-Specific Cleavage of Ribosomal RNA in *Escherichia coli*-Based Cell-Free Protein Synthesis Systems

**DOI:** 10.1371/journal.pone.0168764

**Published:** 2016-12-19

**Authors:** Jurek Failmezger, Robert Nitschel, Andrés Sánchez-Kopper, Michael Kraml, Martin Siemann-Herzberg

**Affiliations:** 1 Institute of Biochemical Engineering, University of Stuttgart, Stuttgart, Germany; 2 Ceqiatec, Costa Rica Institute of Technology, Cartago, Costa Rica; University of Lethbridge, CANADA

## Abstract

Cell-free protein synthesis, which mimics the biological protein production system, allows rapid expression of proteins without the need to maintain a viable cell. Nevertheless, cell-free protein expression relies on active *in vivo* translation machinery including ribosomes and translation factors. Here, we examined the integrity of the protein synthesis machinery, namely the functionality of ribosomes, during (i) the cell-free extract preparation and (ii) the performance of *in vitro* protein synthesis by analyzing crucial components involved in translation. Monitoring the 16S rRNA, 23S rRNA, elongation factors and ribosomal protein S1, we show that processing of a cell-free extract results in no substantial alteration of the translation machinery. Moreover, we reveal that the 16S rRNA is specifically cleaved at helix 44 during *in vitro* translation reactions, resulting in the removal of the anti-Shine-Dalgarno sequence. These defective ribosomes accumulate in the cell-free system. We demonstrate that the specific cleavage of the 16S rRNA is triggered by the decreased concentrations of Mg^2+^. In addition, we provide evidence that helix 44 of the 30S ribosomal subunit serves as a point-of-entry for ribosome degradation in *Escherichia coli*. Our results suggest that Mg^2+^ homeostasis is fundamental to preserving functional ribosomes in cell-free protein synthesis systems, which is of major importance for cell-free protein synthesis at preparative scale, in order to create highly efficient technical *in vitro* systems.

## Introduction

Cell-free protein synthesis systems have emerged as a powerful tool for quick small-scale protein production. Compared with *in vivo* protein expression, these systems have many advantages. For example, cell-free gene expression systems tolerate the incorporation of non-canonical amino acids [1], thus expanding the potential of biological protein synthesis [2–4]. Moreover, the open and modular features of *in vitro* translation systems allow easy automation, direct access to the catalytic system, and high-throughput production [5–7]. Driven by the low productivity of early *in vitro* translation reactions, substantial efforts have been made to understand cell-free translation systems on a metabolic level. For example, several amino acids that were consumed in an expression-independent manner due to the manifold metabolic activities in an *E*. *coli* cell-free extract were identified [8,9]. Furthermore, numerous strategies were developed for supplying energy to the translation reaction [10–14].

By contrast, less attention has been paid to the translation machinery consisting of ribosomal RNA (rRNA), ribosomal proteins and translation factors under *in vitro* conditions. Using high-resolution two-dimensional gel electrophoresis, Schindler et al. investigated the dynamics of certain translation-associated proteins during cell-free protein synthesis [15]. Their study showed that elongation factors were affected by proteolytic decay. However, the stability of ribosomes or rRNA in cell-free protein synthesis systems was not addressed.

Ribosomes are stable structures in living cells [16]. However, they are degraded under certain stress conditions that are typically associated with starvation or slow growth [17–20]. Although it is evident that ribosome degradation in *E*. *coli* occurs at many instances, the factors that trigger this multifaceted phenomenon remain to be elucidated. It was proposed that exposure of intersubunit surfaces might trigger the breakdown of ribosomes by certain endonucleases, resulting in large fragments that are further degraded by exoribonucleases [21]. It was also shown that a substantial percentage of *E*. *coli* ribosomes are degraded as the cells transition from the exponential growth phase to the stationary phase [22]. These reports suggest that growing cells contain ribosome degradation mechanisms.

In order to achieve high product titers, *in vitro* translation systems are designed to mimic the *in vivo* environment of rapidly growing *E*. *coli* cells. Therefore, we investigated whether an *in vitro* translation system is prone to similar degradative processes as its *in vivo* counterpart.

In this study we examined the stability of the *in vitro* translation system during the processing of a cell-free extract (the so-called S30 extract) and *in vitro* translation.

Given the challenge of analyzing a highly complex system consisting of various targets involved in translation, we investigated the integrity of ribosomes by 16S and 23S rRNA analysis. Additionally, we focused on elongation factors and ribosomal protein S1 (RPS1). We found that processing of the S30 extract preserves the *in vivo* stoichiometry between elongation factors and ribosomes. Interestingly, we observed that depending on the *in vitro* reaction conditions, the 16S rRNA is specifically cleaved at the 3´-end near helix 44 resulting in the removal of the anti-Shine-Dalgarno sequence. We also show that the resulting defective 30S ribosomal subunits accumulate in the cell-free translation system. We propose a mechanism where the specific cleavage of 16S rRNA is induced by decreased concentrations of Mg^2+^.

## Results

### Lysate processing does not alter composition of the translation machinery

**Fig 1:**
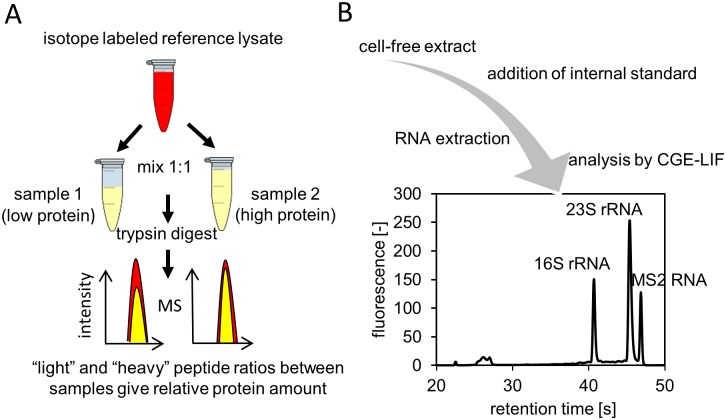
Experimental setup for investigation of the *in vitro* translation machinery. (A) To measure elongation factors from the cell-free extract, samples were mixed with an isotope-labeled cell-extract, which served as an internal reference. (B) rRNA analysis was performed by adding an internal standard (MS2 RNA) to the samples and then performing total RNA extraction. Samples were measured by CGE-LIF and rRNA was quantified based on the known amount of internal standard.

Cell-free translation systems exploiting *E*. *coli* extracts have traditionally relied on the lysate processing strategy primarily described by Pratt and Zubay [23,24]. To date, this process has undergone only minor changes and usually consists of a cell-lysis step, extensive centrifugation to remove any cell debris, a so-called “runoff” reaction which is supposed to activate the translation machinery by disengaging the ribosomes from the mRNAs, a dialysis step, and a final centrifugation step. To systematically analyze the integrity of the translation machinery during lysate processing, we required a strategy that would enable measurement of components involved in translation (Fig 1).

Given that the large number of proteins involved in translation would make a quantitative assessment impractical, we chose to focus on proteins that have previously been shown to be critical for *in vitro* translation systems, specifically EF-Tu, EF-Ts, EF-G and RPS1 [25,26]. We identified two signature peptides representing each elongation factor and RPS1 by MS (Table 1), which were monitored by adding stable isotopic labeled proteins for their relative quantification.

**Table 1:** Signature peptides to monitor elongation factors and RPS1.

Protein	Peptides	m/z	m/z labeled	Retention time [min]
**EF-Tu**	TTLTAAITTVLAK	652.8992	681.4954	48.41
	FESEVYILSK	607.8217	636.4172	34.53
**EF- Ts**	IGVLVAAK	770.5134	806.6341	22.24
	ITDVEVLK	458.7717	479.3411	23.94
**EF-G**	NIGISAHIDAGK	598.3243	623.9115	22.11
	AGDIAAAIGLK	999.568	1043.7325	34.33
**RPS1**	AYEDAETVTGVINGK	783.8901	817.4995	30.28
	GVVVAIDKDVVLVDAGLK	905.5391	946.6768	46.63

**Fig 2:**
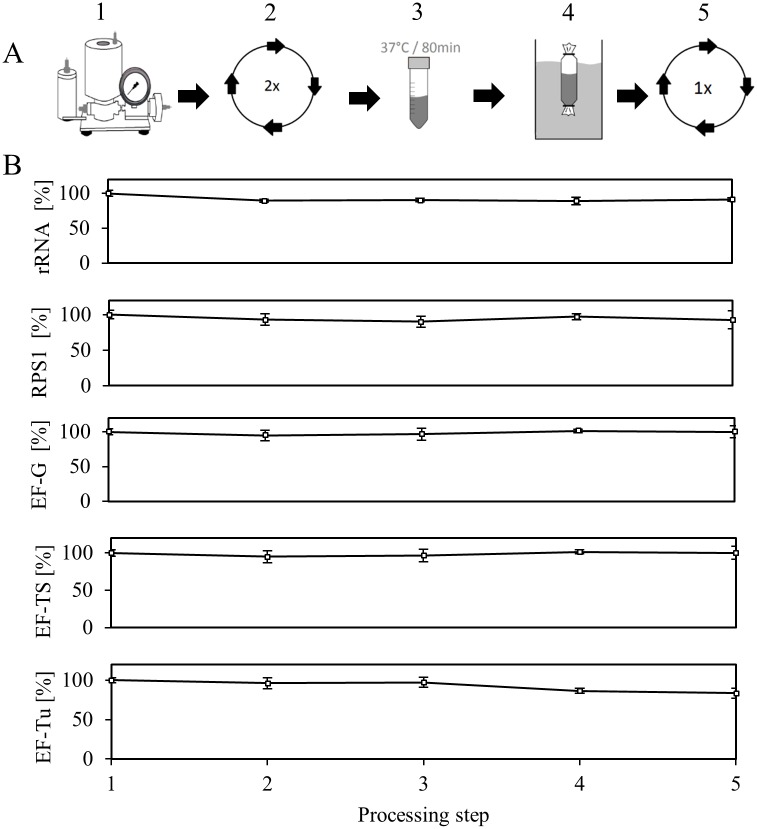
Course of elongation factors, rRNA and RPS1 during lysate processing. (A) Cells were lysed using a high- pressure homogenizer (1); the homogenate was clarified by centrifugation (2); a runoff reaction at 37°C was performed for 80 min (3); the lysate was dialyzed for 4 h (4); the lysate was clarified by a final centrifugation step (5), reprinted with permission by M. Rauter. (B) After each processing step, samples were taken and the level of rRNA (shown as the sum of 16S and 23S rRNA, n = 3, standard deviation ≤ 6%), elongation factors, and RPS1 was measured. Standard deviations consider 3 replicates and 2 signature peptides for each protein.

The integrity and the concentration of ribosomes in the cell-free extract were determined by a previously described rRNA analysis approach, which is based on total RNA extraction and analysis by capillary gel electrophoresis with laser-induced fluorescence detection (CGE-LIF) [27]. As the ribosome mainly consists of rRNA, which also performs the catalytic functions, rRNA degradation is a good measure of ribosome breakdown. By combining proteomics and rRNA analysis we monitored the level of elongation factors and ribosomes during the lysate processing step (Fig 2). Thus, we took samples after each processing step, which were flash frozen in liquid nitrogen and stored at -70°C until analysis. For the analysis of rRNA, samples were processed as described in the Materials and Methods section. Given that the introduced mass shift between samples and reference cell extract created distinct signals for every proteolytic peptide present, we directly compared the peak areas of labeled and unlabeled peptides to deduce the relative amount of proteins in the sample. As the lysate processing is a lengthy procedure, our primary concern was that ribosomes or elongation factors might become unstable over time, which could result in degradation or aggregation and precipitation [28]. The consequence would be a breakup of the given *in vivo* stoichiometry between ribosomes and translation factors (there are approximately 5–6 EF-Tu, 0.2 EF-Ts and 0.8 EF-G molecules per ribosome in fast growing *E*. *coli* [29]). We addressed the runoff procedure, a simple incubation step at 37°C which is generally considered inevitable to obtain a highly active cell-free extract, as the most critical step here. As other labs, we found extensive precipitation during this incubation step [30], the nature of which has not been understood yet. Surprisingly, no decrease in rRNA or any monitored proteins was detected during the runoff procedure (Fig 2B). Moreover, a substantial decrease in the 16S rRNA or the 23S rRNA could not be observed during the lysate processing procedure. Although the cell-free extract possesses RNase activity (see below), this result strongly points to a mechanism that protects ribosomes from breakdown during lysate processing. Among the questioned proteins involved in translation, only EF-Tu showed a 20% decrease, which occurred during the dialysis. Interestingly, longer dialysis steps, for example overnight, resulted in a similar drop in EF-Tu level (data not shown) indicating that EF-Tu might bind to the dialysis membrane.

### MS of elongation factors and RPS1 during *in vitro* translation reaction

**Fig 3:**
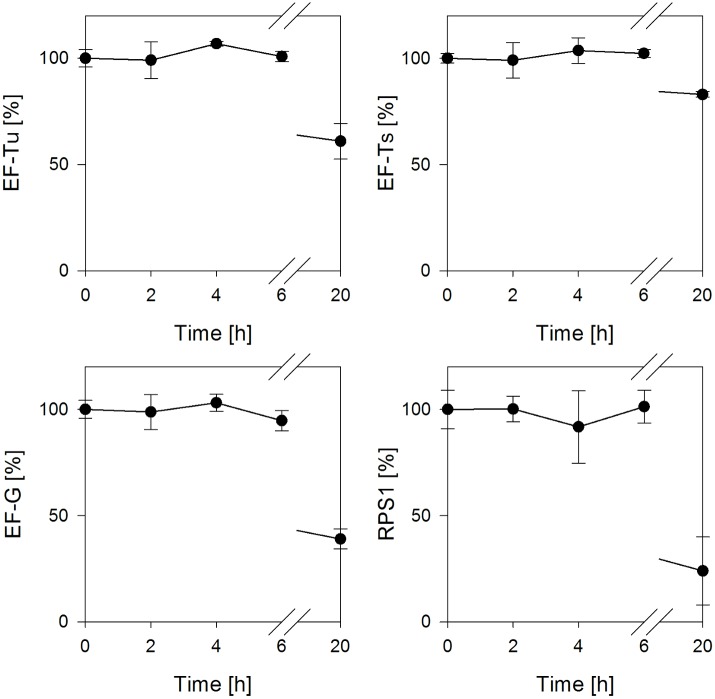
Relative concentrations of elongation factors and RPS1 during the *in vitro* translation reaction. Samples were taken at the denoted time points and proteins were extracted using chloroform/methanol. Analysis of proteins was performed using LC-QTOF-MS. Standard deviations consider 3 replicates and 2 signature peptides for each protein.

To investigate the possible dynamics of the protein composition during an *in vitro* translation reaction, samples were taken and the extracted proteins were subjected to analysis by LC-QTOF-MS. Typical *in vitro* batch reactions run between 2 and 3 h [25,31], however, we chose to monitor proteins for a duration of 6 h and took another sample after a 20 h incubation. Accordingly, this includes a time period when active translation had already collapsed and the ribosome and translation factors might have become substrates for degradation. The data in Fig 3 show the course of elongation factors and RPS1 during the mentioned time. As it can be seen, the concentrations of elongation factors and RPS1 were unaffected during the monitored time of 6 h. Thus, it appears that even after a termination of the translation reaction, proteins involved in translation remained stable for an extended period. Nevertheless, the extended incubation of the cell-free extract for 20 h resulted in a substantial degradation of all monitored proteins demonstrating that elongation factors are susceptible to turnover and denaturation.

### Ribosome degradation during *in vitro* translation

**Fig 4:**
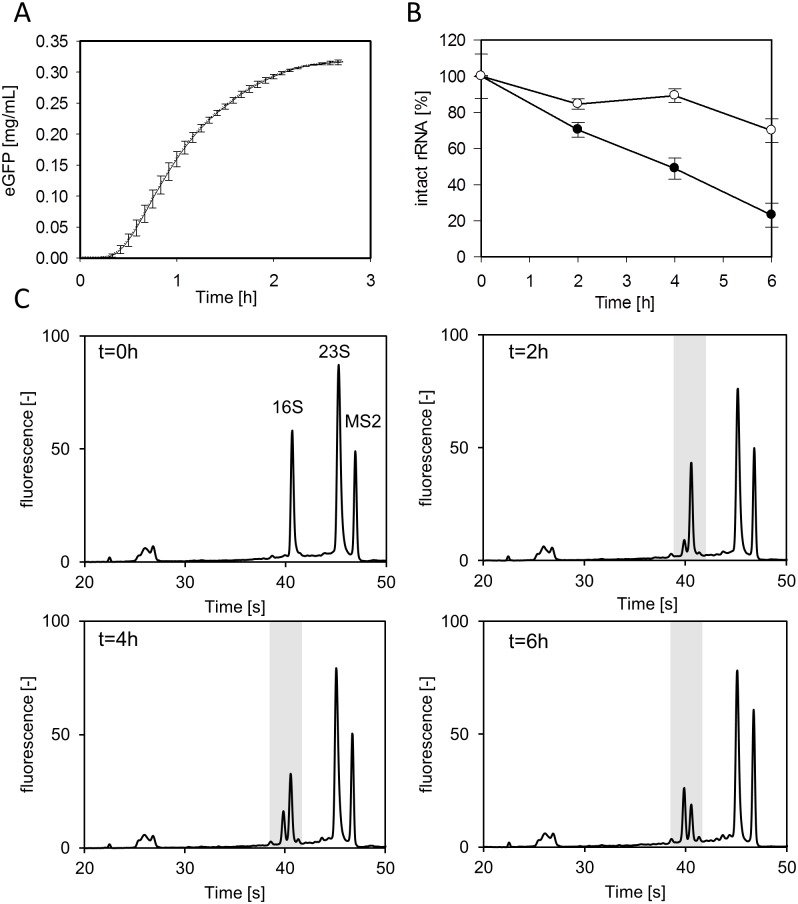
rRNA degradation during the *in vitro* translation reaction. (A) *In vitro* translation reaction of eGFP. Fluorescence emission was continuously recorded using a microplate reader (n = 3). (B) Time course of intact 23S rRNA (○) and 16S rRNA (●) during the reaction (n = 3). Total RNA was extracted and samples were analyzed by CGE-LIF and rRNA was quantified based on the added internal standard (MS2 RNA). C) Electropherograms of analyzed rRNA samples at the denoted time points. The cleaved 16S rRNA fragment accumulates throughout the *in vitro* translation reaction.

Stable RNA such as tRNA and rRNA are only degraded *in vivo* under certain conditions of stress such as nutrient deprivation [16]. As current cell-free translation systems try to mimic an *in vivo* environment, we raised the question of whether ribosomes are stable under *in vitro* reaction conditions. In principle, any mechanisms resulting in ribosome breakdown in living cells should be inherent in cell-free systems. In an attempt to investigate ribosomes in cell-free systems, we performed an *in vitro* translation reaction of eGFP (Fig 4A) and took samples to assay the 16S and 23S rRNA concentration over time (Fig 4B). We found pronounced degradation of the 16S rRNA compared with a moderate decrease in the 23S rRNA concentration. In fact, the amount of intact 16S rRNA continuously decreased, falling to about 20% after 6 h. Surprisingly, the analysis of the respective electropherograms, which are the outcome of the CGE-LIF measurements, revealed that, while the intact 16S rRNA fragment decreased, a slightly shorter peak increased over time (Fig 4C). Hence, we assumed this to be the product of a possible 16S rRNA cleavage. Remarkably, this smaller 16S rRNA fragment accumulated in the *in vitro* translation reaction and was not degraded further. Therefore, after 4 h over 80% and after 6 h over 60% of the total 16S rRNA (sum of intact and cleaved rRNA) was detected. From this experiment, it is evident that: (i) the 16S rRNA of the small ribosomal subunit (30S) is more sensitive to degradation than the 23S rRNA of the 50S subunit; (ii) rRNA degradation is initiated during active translation and continues even after translation has terminated (at this point, a major fraction of 16S rRNA is still intact); (iii) the 16S rRNA is specifically cleaved during degradation to yield a shorter fragment.

To elucidate the mechanism behind the observed degradation process, a screening for potential trigger factors of rRNA breakdown was carried out. Our attempt was to compare the detected 16S rRNA degradation in the running *in vitro* translation systems to the breakdown under certain conditions that have previously been reported to induce rRNA degradation. Accordingly, these conditions should result in an elevated rRNA degradation rate compared with the running system.

**Fig 5:**
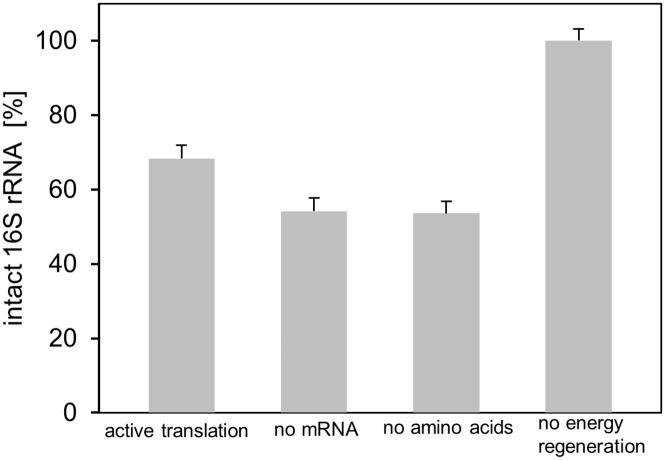
Comparison of 16S rRNA degradation as a result of certain induced conditions. The remaining intact 16S rRNA fraction after a 2 h incubation is shown (n = 3).

For example, it has been shown that free ribosomal subunits are the target of excessive degradation [21]. Thus, we omitted the transcription unit consisting of the plasmid encoding eGFP and the T7 RNA polymerase from the cell-free system, consequently inhibiting ribosomes from assembling into stable translation complexes on the mRNA. In addition, as it is known that carbon starvation triggers ribosome breakdown *in vivo* [16,21], we decided to exclude all amino acids from the system. Moreover, the energy regenerating system consisting of creatine phosphate and creatine kinase was excluded to induce a low energy status. After 2 h of the reaction, we sampled all tested conditions and determined the amount of intact 16S rRNA relative to the beginning amount (Fig 5).

**Fig 6:**
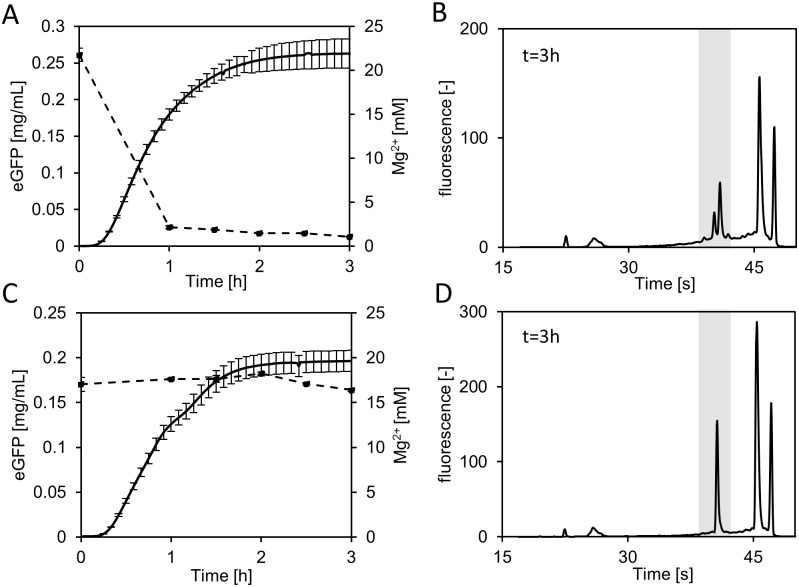
Impact of the Mg^2+^ concentration on degradation of rRNA. (A) Time course of the Mg^2+^ concentration (n = 3, dashed line) during *in vitro* translation of eGFP (solid line) with creatine phosphate for ATP regeneration. (B) Electropherogram of extracted rRNA after a 3 h reaction with creatine phosphate. (C) Time course of the Mg^2+^ concentration (n = 3, dashed line) during *in vitro* translation of eGFP (solid line) with pyruvate for ATP regeneration. (D) Electropherogram of extracted RNA after 3 h reaction with pyruvate shows no signs of rRNA breakdown. Note the different initial Mg^2+^ concentrations for each system, established in a Mg^2+^ optimization step. The error for Mg^2+^ measurements was ≤ 5%. The fluorescence of eGFP was monitored online.

We found slightly more degradation under conditions where either no mRNA was available or amino acids were missing, giving an indication that both conditions favor rRNA degradation, whereas active translation seemed to prevent ribosome degradation to some extent. Interestingly, in the absence of ATP regeneration, no degradation of the 16S rRNA was observed. Synthesis of proteins is highly dependent on energy and requires 4 to 5 molecules of ATP/GTP per peptide bond formation [10]. Common energy regenerating systems, which are necessary to drive *in vitro* translation reactions are based on molecules with a high-energy phosphate bond such as phosphoenolpyruvate or creatine phosphate [10,31]. Those systems lead to the accumulation of inorganic phosphate. Kim et al. reasoned a sequestration of Mg^2+^ ions as the result of accumulating inorganic phosphate as a possible cause for the inhibition of protein synthesis. However, the underlying mechanism was not addressed [31]. Given that we also operated our standard *in vitro* translations system using creatine phosphate and could not detect any ribosome degradation in the sample where creatine phosphate was omitted, we hypothesized that the availability of free Mg^2+^ could induce the observed ribosome degradation. To substantiate this hypothesis, we measured the free Mg^2+^ concentration in a cell-free translation system fueled by creatine phosphate and compared it with a system where we used pyruvate to regenerate ATP. As the usage of pyruvate does not result in accumulation of phosphate [32], we expected a constant level of Mg^2+^ during the reaction. Moreover, we compared the ribosome integrity of both systems after the 3 h reaction. The regeneration of ATP by means of creatine phosphate resulted in a severe drop in Mg^2+^ concentration from 22 to 2 mM within the first hour of the cell-free protein synthesis reaction (Fig 6A). After this fast initial increase, the Mg^2+^ concentration decreased slowly and reached 1 mM at the end of the monitored time span. The respective CGE-LIF electropherogram from extracted RNA after 3 h clearly shows intact and cleaved 16S rRNA (Fig 6B). By contrast, using pyruvate as an energy source resulted in no detectable decrease in Mg^2+^ in the cell-free system (Fig 6C). Furthermore, no cleaved 16S rRNA was observed (Fig 6D). Next, we asked whether ongoing cleavage in the creatine phosphate-containing reaction could be stopped by increasing the Mg^2+^concentration. After 2 h of the *in vitro* translation reaction 20 mM Mg^2+^ was spiked and the sample was further incubated for 1 h. The addition of Mg^2+^ resulted in an 8-fold reduction in 16S rRNA cleavage compared with an unspiked control (S2 Table).

In summary, these data suggest that free Mg^2+^ prevents the degradation of ribosomes in cell-free translation systems, whereas 16S rRNA cleavage is initiated as a consequence of reduced Mg^2+^ concentrations.

### Cleavage of 16S rRNA removes the anti-Shine-Dalgarno sequence

**Fig 7:**
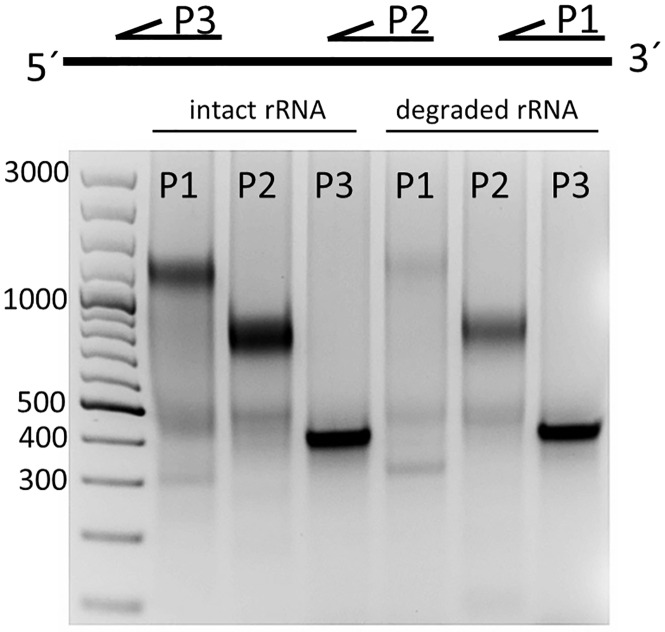
Primer extension analysis of intact and cleaved 16S rRNA. Intact and cleaved 16S rRNA were purified from a 2% agarose gel. Primers specific to regions 1470 to 1491 bp (P1), 895 to 915 bp (P2), 431 to 450 bp (P3) were annealed and cDNA synthesis was performed. RNA was digested and cDNA was visualized on a 1% agarose gel.

In order to comprehensively characterize the observed 16S rRNA cleavage we first examined which end of the 16S rRNA was prone to degradation. Since the 3´-end of the 16S rRNA is part of the anti-Shine-Dalgarno sequence, a pyrimidine-rich sequence that can base pair with the Shine-Dalgarno sequence of the mRNA to initiate translation [33], cleavage of the 3´-end would presumably inactivate the 30S ribosomal subunit. To pursue this idea, intact and cleaved 16S rRNA were purified from agarose gels and a primer extension analysis was carried out using the same amount of rRNA for each sample. Primers covering parts of the whole 16S rRNA were annealed and cDNA was synthesized. Upon digestion of the remaining RNA by RNase A, the cDNA products were analyzed on an agarose gel. In case of the intact 16S rRNA, cDNA products with the sizes expected for all 3 primers were obtained (Fig 7). Strikingly, for the cleaved 16S rRNA no cDNA product for the primer binding to the 1470 to 1491 bp region was detected. Thus, this result gives a strong indication that the cleavage targets the 3´-end of the 16S rRNA aiming to remove the anti-Shine-Dalgarno sequence.

### Identification of the cleavage site

**Fig 8:**
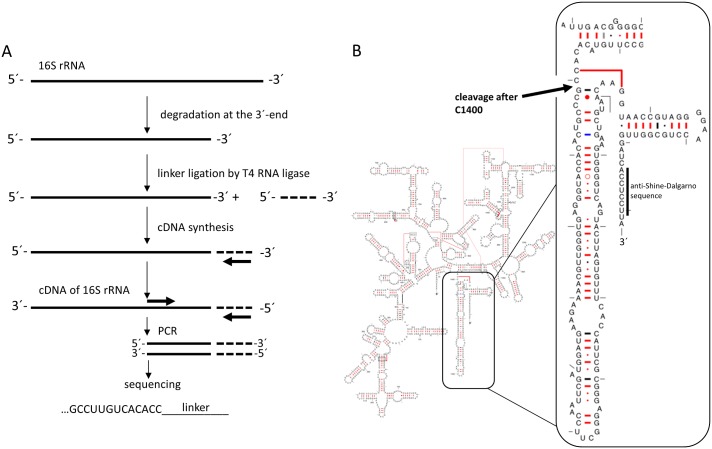
Identification of the 16S rRNA cleavage site. (A) Sequencing of the final PCR products suggests a cleavage after C1400. (B) 16S rRNA secondary stucture [50]. The identified cleavage site is marked with an arrow.

Based on the retention times in CGE-LIF measurements and the known sizes of 16S rRNA, 23S rRNA, and MS2 RNA, we estimated the accumulating cleaved 16S rRNA to have a size in the range of 1401 ± 8 bp. This result reveals that cleavage has to occur somewhere in the region between helices 43 and 44 of the 16S rRNA. To obtain the exact cleavage site, the 16S rRNA cleavage product was purified and a short DNA linker was attached to the shortened 3´-end using T4 RNA ligase. After annealing a primer to the linker, cDNA was synthesized. Next, the generated cDNA was amplified by PCR and the PCR product was sequenced. Sequencing of 3 independently prepared products revealed a single cleavage site between C1400 and G1401, which is in accordance with the estimated size of the cleaved fragment calculated above (Fig 8A). Interestingly, this region (Fig 8B) provides crucial intersubunit bridges that connect the small and big ribosomal subunits. Moreover, Gabashvili et al. suggested this region to be highly dynamic owing to its important function in ribosome association [34].

**Fig 9:**
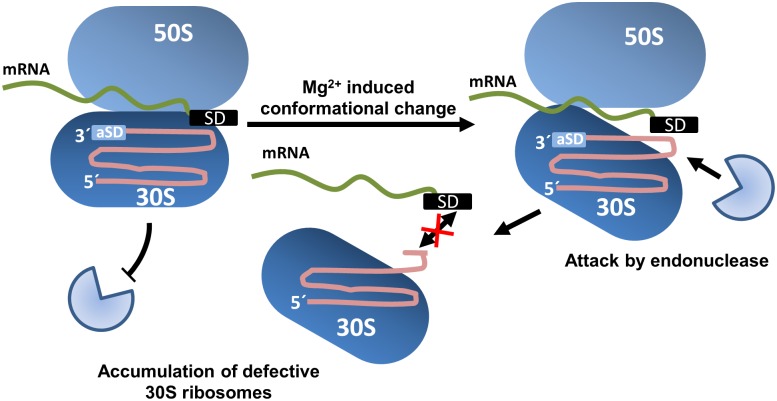
Hypothesis of the observed 16S rRNA degradation. A conformational change in the small ribosomal subunit leads to exposure of helix 44 which is subsequently attacked by an unidentified endonuclease. Cleavage leads to the accumulation of defective 30S ribosomal subunits.

We suggest a model for the observed rRNA breakdown, where stress induced by Mg^2+^ ion starvation results in a conformational change in an already highly dynamic region of the small ribosomal subunit (Fig 9). As a result, helix 44 is exposed and subsequently cleaved by an endoribonuclease. This hypothesis is supported by previous findings: For example, Shenvi et al. showed that the flexibility of the ribosome was directly impacted by Mg^2+^ [35]. It can be hypothesized that our observed cleavage functions as a signal for other nucleases to attack the defective ribosomal subunits for further breakdown.

## Discussion

The present study examined the crucial components involved in translation in cell-free protein synthesis systems. Combining MS and rRNA measurements, we analyzed the dynamics of the elongation factors and ribosomes during the processing of a cell-free extract. We found a slight decrease in EF-Tu during the dialysis step, while other examined compounds such as EF-G, EF-Ts and RPS1 remained unchanged. Moreover, no significant decrease in rRNA was observed. Our major concern was that the commonly applied lysate processing technique would disrupt the *in vivo* stoichiometry of the components involved in translation. However, such a disruption was not observed. It should be noted that, although our data suggest a conserved stoichiometry for *in vitro* systems relative to that of *in vivo* systems, the overall concentration of translation factors in cell-free translation systems is greatly reduced, giving rise to several unfavorable kinetic effects (manuscript in preparation).

To our surprise we discovered severe degradation of 16S rRNA during the cell-free protein synthesis which was fueled by creatine phosphate. The utilization of phosphorylated energy donors for ATP regeneration results in excessive accumulation of inorganic phosphate. There have been speculations that phosphate might inhibit the translation reaction by complexing free Mg^2+^ ions [12,14,31]; however, no causal mechanism has been proposed. Comparison of ribosome stability in two systems that differed in their ability to maintain a Mg^2+^ homeostasis allowed us to link the observed 16S rRNA degradation directly to the availability of free Mg^2+^ ions. This finding was strongly supported by the results of an experiment where the addition of Mg^2+^ to an ongoing degradation process stabilized the remaining intact rRNA. It is known that ribosome decay can be a result of Mg^2+^ starvation [36] and our study completes the picture that Mg^2+^ homeostasis is critical for preventing ribosome degradation in cell-free protein synthesis systems. This is also emphasized by the fact, as shown previously, that recycling of phosphate by the usage of polysaccharide resulted in long lasting *in vitro* translation systems [37,38].

Analysis of ribosome degradation revealed that the 16S rRNA was the main target for breakdown in our system, a phenomenon which has already been observed *in vivo* [18]. Moreover, we found that the 16S rRNA was not fully degraded but accumulated as a specifically cleaved product, which was clearly resolved in CGE-LIF electropherograms. Interestingly, we could not detect the associated smaller fragment, indicating rapid degradation. Further investigations of the cleavage product showed that the 3´-end of the 16S rRNA was heavily affected by the degradation process. In fact, cleavage occurred at helix 44 of the 30S ribosomal subunit. This loss of around 150 bp removes the anti-Shine-Dalgarno sequence and several other important structural features: (i) the anti-Shine-Dalgarno sequence is important for the formation of the mRNA-30S initiation complex; (ii) helix 44 is reported to provide most of the intersubunit bridges [34]. Thus, it follows that these defective ribosomal subunits are unable to assemble into functional ribosomes. A detailed examination revealed a cleavage after C1400. Interestingly, this is only 4 bases downstream from a previously described cleavage site of endonuclease MazF [39]. MazF is part of the toxin-antitoxin system MazEF, which is triggered by the alarmone ppGpp and numerous other stressors [40]. To generate the cell-free extract, we harvested the cells during the early exponential phase. In principle, MazF should be inactive under these conditions. Nevertheless, the results of Vesper at al. support the idea that helix 44 of 16S rRNA is a target of endonucleases. Hence, it can be assumed that helix 44 of the 16S rRNA is a general starting point for ribosome breakdown.

The reason for the accumulation of defective ribosomes in cell-free systems remains uncertain. Using strains lacking specific exoribonucleases that were exposed to glucose starvation, Basturea et al. found accumulation of different rRNA cleavage products. They suggested a general degradation mechanism wherein rRNA is first cleaved by endoribonucleases and the resulting fragments are processed by exoribonucleases [41]. It can be speculated that certain ribonucleases necessary for total rRNA breakdown are only expressed in the cell in response to the changing environmental conditions, and their presence in a cell-extract depends on the physiological state of the cell at the time of harvest; these ribonucleases were not or only in small amounts present in our cell-extract. This hypothesis is supported by the finding that *de novo* protein synthesis is required for ribosome degradation [42].

We identified that reduced concentration of free Mg^2+^ ions is the trigger for 16S rRNA cleavage. Divalent ions are known to encourage the formation of secondary and tertiary structures in RNA [43]. Moreover, Mg^2+^ ions can interact with riboswitches to direct transcription processes by altering mRNA conformations [44]. It is known that ribosome structure is directly influenced by Mg^2+^ ions; ribosomes become less compact at low levels of Mg^2+^ [45], thereby facilitating endoribonucleases to catalyze rRNA breakdown. Most likely, a loss of Mg^2+^ ions in our system induces a conformational change in the 30S ribosomal subunit, exposing helix 44 and allowing an endonuclease to attack. Whether a similar mechanism can be triggered under *in vivo* conditions is a question for future studies.

Our findings contribute to the ongoing research in the field of ribosome decay. As mechanisms and regulatory signals that trigger these degradation processes in *E*. *coli* continue to be elucidated [21,42], our findings lend further support to the emerging picture that exposure of rRNA-rich regions initiates ribosomal degradation. Moreover, we showed that Mg^2+^ homeostasis is the key parameter for maintaining functional ribosomes in cell-free protein synthesis systems.

## Materials and Methods

### Mass spectrometry of elongation factors

Liquid chromatography-quadrupole time-of-flight-MS (LC-QTOF-MS) analysis was performed with the same conditions as described earlier for peptide mapping of intracellular extracts, using an Agilent 6540 Accurate-Mass Quadrupole-Time of Flight instrument coupled to an Agilent 1260 Infinity Bio-inert HPLC system, equipped with an Aeris-PEPTIDE (3.6 μ XB-C18 150 × 2.1 mm) column and pre-column from Phenomenex, where the mass spectrometer was configured to automatic acquire fragmentation spectra (MS/MS) for peptides signals [46]. Raw data were processed using the Mass Hunter Workstation Software (Ver.B.05.519.0, Agilent Technologies). The acquired MS/MS spectra were *de novo* sequenced and aligned against an *E*. *coli* K12 database (4042 sequences) using PEAKS Studio 7 search engine (Bioinformatics Solutions, Waterloo, Canada) with the following parameters: enzyme: trypsin; parent mass error tolerance: 10 ppm; fragment mass error tolerance: 0.01 Da; max. missed cleavages: 100. The false discovery rate, as calculated by decoy database search, was 1%. Only peptides with mass accuracy better than 5 ppm, and five identified a/b fragments were considered as signature peptides. The mass spectrometry proteomics data have been deposited to the ProteomeXchange Consortium via the PRIDE [47] partner repository with the dataset identifier PXD004703 and 10.6019/PXD004703.

Two unique peptides per protein were chosen for analysis of EF-Tu, EF-Ts, EF-G and RPS1.

Stable isotopic labeled cell extract was generated by *E*. *coli* culture in the presence of D-Glucose-^13^C_6_. Samples were mixed with this isotope labeled reference lysate before in solution digestion for relative quantification of proteins. Extracted ion chromatograms of labeled and unlabeled peptide´s molecular ions were matched by predicted m/z and identical retention time. Peak area ratios of heavy and light peptides were determined using the Quantitative Analysis Software and used for relative quantification (Ver.B05.01, Agilent Technologies).

### In-solution digestion of proteins

Proteins (100 μg) in 6 M urea solution were reduced by DTT for 1 h at room temperature followed by alkylation for 1 h in the dark. Samples were diluted with 50 mM Tris-HCL, pH 8.0. Digestion was initiated by adding of 2 μg trypsin (Promega) to the samples and incubating them at 37°C over-night. Samples were brought to a final concentration of 5% (v/v) acetonitrile and 1% (v/v) formic acid before analysis by LC-QTOF-MS.

Prior to in-solution digestion, samples from *in vitro* translation reactions were purified using methanol/chloroform extraction.

### Generation of stable isotope-labeled cell-extract

An overnight culture of *E*. *coli* A19 was cultivated in the presence of uniformly-labeled D-Glucose-^13^C_6_ (Silantes, Germany) in minimal media containing 1 mM methionine. Cells were harvested by centrifugation at 5000 *x*g for 20 min at 4°C. The cell pellet was resuspended in S30 buffer (for composition, see below) and cells were lysed using a high pressure homogenizer. The isotope-labeled cell-extract was aliquoted and stored at -70°C.

### Cell-free extract preparation

*E*. *coli* A19 was cultivated batchwise on 2 × YTPG medium in a 30 L bioreactor. Cells were harvested during the early exponential phase by pumping through a plate heat exchanger, where the biomass was rapidly chilled to 4°C and by centrifugation at 17,000 *x*g in a CEPA High Speed Centrifuge. The subsequent cell-free extract preparation was carried out according to Liu [23] with some modifications. Briefly, 1 mL of S30 buffer (14 mM magnesium acetate, 60 mM potassium acetate, 10 mM Tris, pH 8.0, 2 mM DTT) was added per gram of cell paste. The cell suspension was lysed by two passes through a high-pressure homogenizer (EmulsiFlex-C5, Avestin, Canada) at 12,000 kPa. The cell debris was removed by two centrifugation steps at 30,000 *x*g for 30 min at 4°C each. A run-off reaction was performed by incubating the cell-free extract for 80 min at 37°C on a shaker. After the run-off, the cell-free extract was dialyzed against a 100-times larger volume of S30 buffer for 4 h at 4°C. The cell-free extract (total protein concentration 37.5 mg/mL as determined by Pierce BCA protein assay) was finally centrifuged at 4000 *x*g for 20 min at 4°C. The extract was aliquoted, frozen in liquid nitrogen, and stored at -70°C.

### Cell-free expression

The standard reaction mixture in a total volume of 250 μL consisted of the following components: 80 mM HEPES-KOH (pH 8.0), 1.2 mM ATP, 1 mM GTP, CTP and UTP, 2 mM DTT, 90 mM potassium glutamate, 20 mM ammonium glutamate, 18 mM magnesium glutamate, 34 μg/mL folinic acid, 1 mM 20 amino acids, 2% PEG (8000), 100 mM creatine phosphate, 240 μg/mL creatine kinase, 3 U/ μL T7 RNA Polymerase (Roche Diagnostics, Mannheim, Germany), 15 μg/mL DNA (pJOE4065.2 was kindly provided by J. Altenbuchner, IIG, University of Stuttgart), 24% (v/v) of S30 extract. In reactions where pyruvate was used, 100 mM pyruvate, 0.3 mM NAD, and 0.26 mM CoA were added. In reactions analyzed by MS, PEG was not added.

Production of eGFP was monitored online by fluorescence detection (excitation filter 485 nm, emission 520 nm) in a Synergy 2 plate reader (Biotek Instruments, USA) at 37°C.

### RNA extraction and rRNA analysis

Total RNA extraction from the cell-free extract was performed as described by Reddy [48] and Hardiman [27] with the following modifications.

Briefly, samples from the cell-free extract or *in vitro* translation reactions were diluted and 20 μL were added to tubes containing 5 μL DEPC and internal standard (MS2 RNA, Roche Diagnostics, Mannheim, Germany) followed by addition of 500 μL of buffer (10 mM Tris-HCl (pH 8.0), 10 mM NaCl, 1 mM sodium citrate and 1.5% (w/v) SDS). The samples were mixed with 250 μL ice-cold NaCl (saturated solution) and incubated on ice for 10 min. The derived protein-SDS-DNA precipitate was collected by centrifugation (20,000 *x*g, 20 min, 4°C) and 500 μL of clear supernatant was transferred to fresh tubes. RNA precipitation was induced by addition of 1 mL ice-cold ethanol (100%) and incubation of the samples at -70°C for 1 h. The precipitated RNA was collected by centrifugation (20,000 *x*g, 30 min, 4°C) and washed with 1 mL ice-cold ethanol (70%). The supernatant was removed and the rRNA-containing pellets were dried in a vacuum centrifuge and stored at -70°C.

All RNA measurements were performed using an Agilent 2100 Bioanalyzer (Agilent Technologies, Palo Alto, USA) according to the manufacturer’s instructions. Prior to RNA analysis, RNA-containing pellets were resuspended in RNase-free water (0.1% DEPC in ddH_2_O, incubated at 37°C overnight and autoclaved) and incubated at 65°C for 5 min to aid resolubilization. Quantification of distinct rRNA species was performed on the basis of the added internal standard.

### Determination of Mg^2+^ concentration

The quantitative measurement of Mg^2+^ was performed as described in [31] using a magnesium assay kit (Abnova, Taiwan) according to the manufacturer’s instructions.

### RNA gel electrophoresis

RNA gel electrophoresis was performed according to [49]. Briefly, RNA samples were mixed with formamide (60% (v/v) final concentration) and denatured at 65°C for 5 min. Samples were run on a 2% agarose gel and RNA was visualized by GelRed^™^(Biotium, USA) staining.

### Primer extension and cDNA analysis

Intact and degraded rRNA were purified from agarose gels using the Zymoclean^™^ Gel RNA Recovery Kit (Zymoresearch, USA) and annealed to primers specific to regions of the 16S rRNA. Primer 1 (1470–1491): 5′-caccccagtcatgaatcacaaa-3′; primer 2 (978–997): 5´-atgtcaagaccaggtaaggt-3´; primer 3 (432–451): 5´-tcctccccgctgaaagtact-3´. cDNA synthesis was performed by reverse transcriptase (SuperScript^®^IV, life technologies, USA) according to the manufacturer's instructions. To remove the RNA, 1 μL of RNase A was added and the samples were incubated for 20 min at 37°C. Samples were loaded on a 1% agarose gel and cDNA was visualized by GelRed™(Biotium, USA) staining.

### Identification of the rRNA cleavage site

A linker consisting of a set of randomly designed oligonucleotides (5-P´-cacggcgcaataccaccacactaccggcgtccaccataccttcgatattcgcgaccactctctcattagc-3-P´) was ligated to the 3′-end of the degraded rRNA fragment using T4 RNA ligase (Thermo Scientific) according to the manufacturer's instructions. cDNA synthesis was accomplished by annealing a primer (5′-gctaatgagagagtggtcgc-3′) to the 3′-end of the linker. The generated cDNA fragment was partly amplified by PCR using oligonucleotides 5´-tgcaacgcgaagaaccttac-3´ and 5´-gctaatgagagagtggtcgc-3´ and subsequently sequenced and mapped against the 16S rRNA.

## Supporting Information

S1 Table Calculation of relative protein amounts during cell-free extract processing. (PDF)

S2 Table Increase in the ratio of cleaved to intact 16S rRNA after 3 h of in vitro translation reaction with and without spiking of 20 mM Mg^2+^. (PDF)
