# Supplementary material for: Site-Specific Cleavage of Ribosomal RNA in Escherichia coli-Based Cell-Free Protein Synthesis Systems
Source: PLoS One. 2016 Dec 19;11(12):e0168764. doi: 10.1371/journal.pone.0168764 (PMC5167549; doi:10.1371/journal.pone.0168764)

S1 Table. Calculation of relative protein amounts during cell-free extract processing.

Processing step	EF-Tu			EF-Ts			EF-G			RPS1		
	ratio peak areas unlabeled/labeled	relative amount [%]	rel. SD [%]	ratio peak areas unlabeled/labeled	relative amount [%]	rel. SD [%]	ratio peak areas unlabeled/labeled	relative amount [%]	rel. SD [%]	ratio peak areas unlabeled/labeled	relative amount [%]	rel. SD [%]
1	5.48	100.00	3.28	4.94	100.00	4.77	4.28	100.00	4.23	5.62	100.00	5.87
2	5.21	94.95	6.96	4.73	95.69	6.73	4.01	93.82	7.79	5.21	90.67	8.13
3	5.20	94.74	6.45	4.87	98.55	7.78	4.15	97.11	8.49	5.07	88.22	7.77
4	4.65	84.80	3.34	5.06	102.27	6.39	4.44	103.74	2.62	5.47	95.29	4.17
5	4.48	81.62	6.37	4.99	100.90	7.19	4.31	100.73	8.58	5.22	90.82	12.62

Ratio of peak areas of unlabeled to labeled petides give relative protein amount in the sample.

Unlabeled peptide FESEVYILSK (EF-Tu)

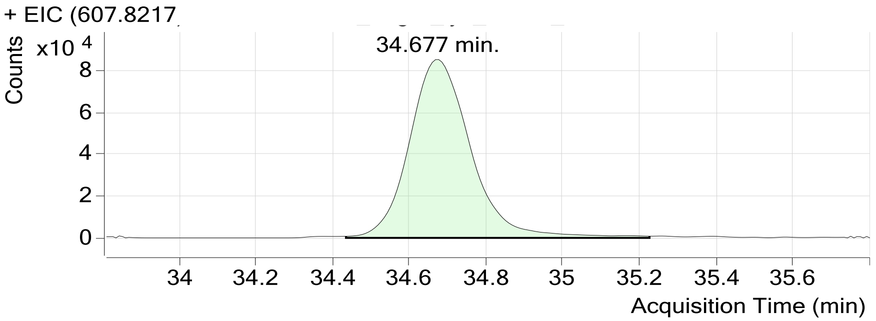

Labeled peptide FESEVYILSK (EF-Tu)

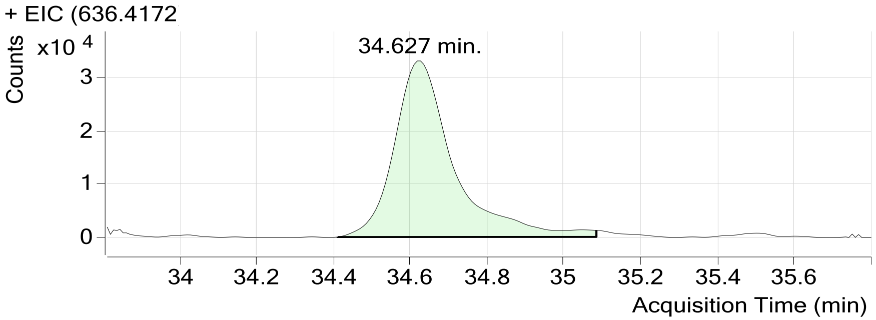

Supplement: S1 Table — (PDF) [file pone.0168764.s001.pdf]
