# Supplementary material for: Site-Specific Cleavage of Ribosomal RNA in Escherichia coli-Based Cell-Free Protein Synthesis Systems
Source: PLoS One. 2016 Dec 19;11(12):e0168764. doi: 10.1371/journal.pone.0168764 (PMC5167549; doi:10.1371/journal.pone.0168764)
Supplement: S2 Table — (PDF) [file pone.0168764.s002.pdf]

S2 Table: Increase in the ratio of cleaved to intact 16S rRNA after 3 h of in vitro translation reaction with and without spiking of 20 mM Mg^{2+}.

	Ratio of cleaved to intact 16S rRNA		
Reaction conditions	2h reaction time	3h reaction time	Relative increase [%]
No Mg^{2+} spike	0.23±0.03	0.35±0.06	51
Mg^{2+}spike	0.18±0.02	0.19±0.02	6

The peak areas of cleaved and intact 16S rRNA were determined after 2 and 3 h by CGE-LIF measurements and are shown as ratios. Addition of Mg^{2+} reduced the cleavage.
